# Effects of edaravone dexborneol on neurological function and serum inflammatory factor levels in patients with acute anterior circulation large vessel occlusion stroke

**DOI:** 10.1515/tnsci-2022-0312

**Published:** 2023-10-12

**Authors:** Xiaohong Hu, Zhenhong Qian, Jianhui Chen, Mingsheng Chen, Wenying Zhong, Chaoxiong Shen, Zhizhou Hu, Rongtong Li

**Affiliations:** Neurology Department, Longyan First Affiliated Hospital of Fujian Medical University, No. 105, Jiuyi North Road, Longyan, 364000, China; Neurology Department, Liancheng County Hospital, No. 1, Miaoqian Road, Liancheng, 366200, China; Emergency Department, Longyan First Affiliated Hospital of Fujian Medical University, No. 105, Jiuyi North Road, Longyan, 364000, China

**Keywords:** anterior circulation large vessel occlusion stroke, edaravone dexborneol, neurological function, thrombectomy

## Abstract

The goal of this study is to evaluate and analyze the effects of edaravone (EDV) dexborneol on neurological function and serum inflammatory factor levels among patients with acute anterior circulation big artery blockage stroke. A total of 142 patients with acute anterior circulation large vessel occlusion (LVO) were randomly allocated to the study group (69 patients) or the control group (73 patients). In the study group, patients were treated with 37.5 mg EDV dexborneol twice a day for 10–14 days, based on the control group. The primary efficacy outcome was the National Institutes of Health Stroke Scale score change from baseline to 90 days and the proportion of modified Rankin Scale (mRS)score ≤1 at 90 days after randomization. The secondary outcome included the decrease in inflammatory factors at 14 days. The primary safety outcome was the incidence of hemorrhagic transformation assessed according to Heidelberg bleeding classification within 7 days. A higher percentage of patients with HIHSS score ≤5 at 90 days in the EDV dexcamphorol group was observed than in the control group (75.36% vs 64.38%; *P* = 0.015). A higher percentage of patients with mRS score ≤1 at 90 days in the EDV dexcamphorol group was observed than in the control group (63.77% vs 50.68%; *P* = 0.012). After treatment, the levels of IL-6 and hs-CRP were significantly lower following treatment and compared to the control group (*P* < 0.05). In patients receiving the EDV dexborneol group, a significantly decreased risk of radiographic intracranial hemorrhage was found compared with the control group (20.29% vs 39.73%; *P* = 0.0006). In conclusion, EDV dexborneol can improve the clinical outcomes of patients with acute anterior circulation LVO stroke, which can be used as an effective supplement to thrombectomy therapy.

## 1 Introduction

Cerebrovascular disease is characterized by high incidence rate, high mortality, high disability rate, and high recurrence rate. Since 2008, it has surpassed tumor and cardiovascular disease and became the first cause of death for urban and rural residents in China. Large hemispheric infarction (LHI) is a critical type of cerebral infarction with higher disability and mortality. The incidence rates of LHI account for 2–8% of all acute ischemic stroke (AIS). Research shows that the mortality rate of large vessel occlusion (LVO) in the anterior circulation is as high as 26% [1]. In patients with conservative treatment, the mortality rate of LHI is as high as 40–80%, seriously endangering human health. Within the time window of onset, thrombolysis and thrombectomy, especially endovascular thrombectomy, are currently the most effective treatments for AIS with LVO [2,3]. However, meta-analysis shows that the ineffective reperfusion rate after mechanical thrombectomy in patients with AIS with LVO is about 54% [2]. Therefore, the Stroke Treatment Academic Industry Roundtable (STAIR) X acknowledges that neuroprotective agents can and should be developed in conjunction with thrombolysis.

Edaravone (EDV), a free radical scavenger developed in Japan, is widely used to treat AIS within 24 h of onset [4,5,6]. Borneol, a terpene and bicyclic organic compound, has shown anti-inflammatory activities and protective effects against cerebral ischemia/reperfusion injury [7]. EDV dexborneol is a national Class I innovative drug, which has been launched in China in 2020. EDV dexborneol is a newer multi-target neuroprotective agent, which has been designed and implemented in accordance with the STAIR principle [8,9].

Previous preclinical studies have confirmed that EDV dexborneol inhibits apoptosis of neurons, glial cells, and vascular endothelial cells by clearing free radicals and reducing inflammatory cascade reactions, showing a protective effect on the entire neurovascular unit, significantly alleviating nerve damage in the reperfusion animal model. In a phase II clinical trial involving over 400 patients with ischemic stroke, the middle dose group of EDV dexborneol (37.5 mg) showed better efficacy and safety [10]. The TASTE trial (Treatment of Acute Ischemic Stroke with Edaravone Dexborneol) was a phase III, randomized, double-blind, parallel, comparative study that enrolled 1,200 patients and showed that EDV dexborneol significantly increased the proportion of functional independence at day 90 in patients with AIS [11].

In the context of the above research, this study used EDV dexborneol to cure sufferers with acute ischemic stroke to explore the therapeutic effect of EDV dexborneol on patients with anterior circulation macrovascular occlusion. In this study, a total of 142 cases of acute anterior circulation macrovascular occlusion were collected. On the basis of standardized treatment (the control group), we added EDV dexborneol (the treatment group) to observe the decrease in inflammatory indicators (IL-6, CRP), bleeding transformation, and clinical outcomes at 90 days. The results are now reported as follows.

## 2 Methods and methods

### 2.1 Study design

A total of 142 patients with anterior circulation LVO stroke admitted to our hospital from October 2021 to September 2022 were retrospectively analyzed and randomly divided into the EDV dexborneol treatment group and the control group. Inclusion criteria: (1) patients with cerebral infarction admitted to our hospital within 48 h of onset; (1) age ≥18 years old; (3) before the onset of this disease, modified Rankin Scale (mRS) ≤2; (4) patients with anterior circulation cerebral infarction who was diagnosed as cerebral infarction through cranial computer tomography (CT) or magnetic resonance imaging (MRI) examination. All enrolled patients underwent computer tomography angiography examination, which indicated LVO. LVO was defined as an occlusion in the internal carotid artery, the M1 or M2 branches of the middle cerebral artery that correlated with the clinical symptoms [12].

Exclusion criteria were as follows: (1) cranial MRI or CT examination of cerebral infarction area does not meet the diagnosis of large area cerebral infarction; (2) patients who are infected or using antibiotics during the admission; (3) combined severe organ failure (heart, liver, and kidney failure); (4) patients with moderate or above anemia, coagulation dysfunction, or other serious blood system diseases; (5) patients with severe endocrine disorders; (6) patients with combined autoimmune system diseases; (7) patients with concomitant malignant tumors; and (8) patients or patient guardians who are unwilling to participate in the project. Randomization was performed via a web-based mobile phone app or computer. Patients were assigned a random serial number according to the time they were enrolled. All trial personnel and patients were unaware of the treatment assignment.

### 2.2 Treatment procedures

All enrolled patients receive standardized diagnosis and treatment in accordance with the “Guidelines for the Diagnosis and Treatment of Acute Ischemic Stroke 2018” [13]. Patients who are within the thrombolysis time window, have no contraindications for treatment, and have the consent of the patient or family members were received alteplase thrombolysis treatment. The alteplase was administered according to the dosage regimen approved in Japan (10% of a dose [0.9 mg/kg] of alteplase was given by rapid intravenous injection; the rest was given by intravenous infusion for 1 h) [14]; patients with arterial thrombectomy indications are strictly evaluated and given thrombectomy treatment with the consent of the patient or their family, while also receiving standardized treatment such as antiplatelet aggregation, lipid metabolism regulation, and bedside rehabilitation. The treatment group was treated with a combination of EDV dexborneol on the basis of the control group. The usage of EDV dexborneol： EDV dexborneol for injection (Nanjing Xiansheng TECO Pharmaceutical Co., Ltd., specification: 5 ml: 10 mg (edaravone): 2.5 mg (dexborneol), national drug approval number: H20200007) intravenous infusion treatment, 15 ml each time (containing edaravone 30 mg, dexborneol 7.5 mg), 2 times per day. When in use, add it to 100 ml of normal saline to dilute it, and then infuse it intravenously.

### 2.3 Observational indicators

(1) The two groups were evaluated for neurological damage using the National Institutes of Health Stroke Scale (NIHSS) score on admission, 10–14 days of treatment, 30 days of treatment, and 90 days of treatment, with a total score of 42 points. The higher the score, the more severe the neurological damage. The degree of disability was evaluated using the mRS on admission, 10–14 days, 30 days, and 90 days of treatment. The mRS score was 0–6 points, and the higher the score, the poorer the patient’s quality of life. Compare the proportion and 95% confidence interval of patients with mRS ranging from 0 to 1 at different time points between the EDV dexacamphor treatment group and the control group. The NIHSS score and mRS score were completed by two neurologists who were not aware of the study. If there were any objections to the score, it was evaluated by a third experienced neurologist. The primary safety outcome was the incidence of hemorrhagic transformation (HT) assessed according to the Heidelberg bleeding classification within 7 days.
(2) Inflammatory factor monitoring: all patients were tested for serum IL-6 and CRP on the day of admission, 5–7 days, and 10–14 days by abdominal blood sampling of 5 mL × 2 of peripheral venous blood. Peripheral phlebo blood was put in an anticoagulant tube for 1.5 h and centrifuged with a centrifuge (Hettich, Germany, Rotofifix 32A) for 10 min at a velocity of 3,000 rpm; separate the supernatant and save it for later use. The density of serum IL-6, CRP, in the two groups of sufferers pretherapy and posttherapy is tested by enzyme-linked-immunosorbent serologic assay means to test plasma-specific viscosity before and after treatment in the two groups. The kits are purchased from Wuhan Boster Company, and the operating routines are rigorously based on the specifications of the reagents and instruments.

### 2.4 Statistical analysis

The data are calculated using the SPSS 20.0 system, and the baseline characteristics between the groups were compared. Categorical variables were presented as counts (percentages) and compared using the chi-square test. Continuous variables were presented as mean ± standard deviation if normally distributed and as median and interquartile range if non-normally distributed. *P* < 0.05 is considered statistically significant.


**Ethical approval:** The research related to human use has been complied with all the relevant national regulations, institutional policies and in accordance with the tenets of the Helsinki Declaration, and has been approved by the authors’ institutional review board or equivalent committee. This study has been reviewed and approved by the Medical Ethics Association of our hospital (Ethics Review Number: 2021-10-26).
**Informed consent:** Informed consent has been obtained from all individuals included in this study.

## 3 Results

### 3.1 Clinical data

In the treatment group, there were 45 men and 24 women, aged 65.8 ± 11.0 years, 53 patients with hypertension, 17 patients with diabetes, 8 patients with coronary heart disease, 20 patients with atrial fibrillation, 22 patients with smoking, 14 patients with alcohol consumption, and 11 patients with previous stroke history. There were 26 patients with internal carotid artery occlusion, 39 patients with middle cerebral artery occlusion, 4 patients with internal carotid artery + middle cerebral artery occlusion, 22 patients who underwent thrombolysis, and 39 patients who underwent thrombectomy. The NIHSS score at admission was 10.0 ± 6.1, and the mRS score was 3.9 ± 1.3; in the control group, there were 44 males and 29 females, aged 66.6 ± 12.7 years, 44 patients with hypertension, 20 patients with diabetes, 11 patients with coronary heart disease, 23 patients with atrial fibrillation, 25 patients with smoking, 15 patients with alcohol consumption, and 16 patients with previous stroke history. There were 21 patients with internal carotid artery occlusion, 48 patients with middle cerebral artery occlusion, 4 patients with internal carotid artery + middle cerebral artery occlusion, 20 patients who underwent thrombolysis, and 40 patients who underwent thrombectomy. The NIHSS score at admission was 10.6 ± 6.4, and the mRS score was 4.0 ± 1.2. The baseline data of age, gender, NIHSS score at admission, and mRS score at admission between the two groups are shown in Table 1. There was no statistically significant difference in various indicators between the two groups of patients (*P* > 0.05).

**Table 1:** Baseline characteristics

Characteristics	Treatment group (*n* = 69)	Control group (*n* = 73)	*P* value
Age	65.8 ± 11.0	66.6 ± 12.7	0.857
Male, *n* (%)	45 (65.2%)	44 (60.2%)	0.741
Hypertension, *n* (%)	53 (76.8%)	44 (60.2%)	0.258
Diabetes, *n* (%)	19 (24.6%)	19 (27.3%)	0.894
Coronary heart disease, *n* (%)	8 (11.5%)	11 (15.1%)	0.815
Atrial fibrillation, *n* (%)	20 (28.9%)	23 (31.5%)	0.874
Smoking, *n* (%)	22 (31.9%)	25 (34.2%)	0.852
Alcohol consumption, *n* (%)	14 (20.2%)	15 (20.5%)	0.926
Stroke history, *n* (%)	11 (15.9%)	21 (28.7%)	0.512
Internal carotid artery occlusion, *n* (%)	26 (37.7%)	20 (27.4%)	0.428
Middle cerebral artery occlusion, *n* (%)	39 (56.5%)	48 (65.7%)	0.365
Internal carotid artery + middle cerebral artery occlusion, *n* (%)	4 (5.8%)	4 (5.5%)	0.999
Thrombolysis, *n* (%)	22 (31.9%)	20 (27.3%)	0.682
Thrombectomy, *n* (%)	39 (56.5%)	40 (54.8%)	0.903
NIHSS score at admission	10.0 ± 6.1	10.6 ± 6.4	0.952
mRS score at admission	3.9 ± 1.3	4.0 ± 1.2	0.935

### 3.2 Comparison of admission test indicators between two groups

The comparison of examination indicators between the treatment group and the control group at admission is shown in Table 2. There was no significant difference in blood WBC, blood sugar, CHOL, LDL-C, HDL, UA, HCY, IL-6, CRP, and other test indicators between the two groups of patients (*P* > 0.05).

**Table 2:** Comparison table of inspection indicator

Project	Treatment group	Control group	*P* value
WBC (10 × 9/L)	9.725 ± 3.289	10.157 ± 3.884	0.776
GLU (mmol/L)	7.243 ± 3.095	8.055 ± 3.27	0.798
CHOL (mmol/L)	4.671 ± 1.326	4.453 ± 1.137	0.882
LDL-C (mmol/L)	3.004 ± 0.846	2.7866 ± 0.8217	0.699
HDL (mmol/L)	1.1036 ± 0.3124	1.0666 ± 0.2409	0.925
UA (μmol/L)	350.7 ± 113.2	327.7 ± 125.6	0.941
HCY (μmol/L)	10.25 ± 9.5	11.456 ± 5.956	0.938
IL-6 at admission (pg/mL)	33.6 ± 57.9	35.4 ± 99.5	0.868
CRP at admission (mg/L)	27.23 ± 41.44	25.24 ± 37.27	0.839

### 3.3 Comparison of changes in NIHSS between two groups

There was no significant difference in NIHSS scores between the two groups of patients upon admission. As the treatment, there was a significant difference in the magnitude of the decrease in NIHSS scores between the study group and the control group, with significant statistical significance (*P* < 0.05). Moreover, the magnitude of the decrease in NIHSS scores between the treatment group was consistently higher than that of the control group. The proportion of NIHSS scores ≤5 in the treatment group and the control group continued to increase with the progress of treatment, and the proportion of NIHSS scores ≤5 in the study group was consistently higher than that in the control group, with significant statistical significance (*P* < 0.05).

Further analysis showed that, compared with the thrombolysis control group, the treatment group showed a greater decrease in NIHSS, with significant statistical significance (*P* < 0.05). The proportion of patients with an NIHSS score of ≤5 reached 75%, indicating that EDV dexborneol can be used as an effective supplementary treatment after thrombolysis. The comparison between the thrombectomy treatment group and the thrombectomy control group showed the greatest decrease in NIHSS after treatment, with a proportion of 76.92% having an NIHSS score of ≤5, indicating a statistically significant difference (*P* < 0.05). This indicates that EDV dexborneol can serve as an effective supplement for thrombectomy treatment. The results are shown in Table 3.

**Table 3:** Comparison table of the NIHSS scores

Group	The magnitude of NIHSS score decrease			Proportion of the NIHSS score of 5 points, *n* (%)		
	14 days	30 days	90 days	14 days	30 days	90 days
Treatment group (*N* = 69)	5.13 ± 5.29	6.62 ± 5.57	8.22 ± 5.69	42 (60.87%)	46 (66.67%)	52 (75.36%)
Control group (*N* = 73)	3.26 ± 4.00	4.34 ± 4.00	5.83 ± 4.17	38 (52.05%)	42 (57.53%)	47 (64.38%)
*P* value	0.005	0.036	0.001	0.028	0.031	0.015
Thrombolysis treatment group (*N* = 24)	7.55 ± 5.52	9.48 ± 5.19	11.1 ± 5.66	14 (58.33%)	17 (70.83%)	18 (75.00%)
Thrombolysis control group (*N* = 21)	2.57 ± 3.31	3.76 ± 3.50	4.90 ± 3.80	11 (52.38%)	12 (57.14%)	13 (61.90%)
*P* value	0.0006	0.003	0.001	0.045	0.033	0.018
Thrombectomy treatment group (*N* = 39)	7.33 ± 5.17	9.38 ± 5.00	10.94 ± 4.71	21 (53.85%)	26 (66.67%)	30 (76.92%)
Thrombectomy Control group (*N* = 40)	3.8 ± 4.20	5.25 ± 4.10	7.05 ± 4.48	19 (47.50%)	22 (55.00%)	25 (62.50%)
*P* value	0.013	0.011	0.025	0.036	0.010	0.004

**Figure 1:**
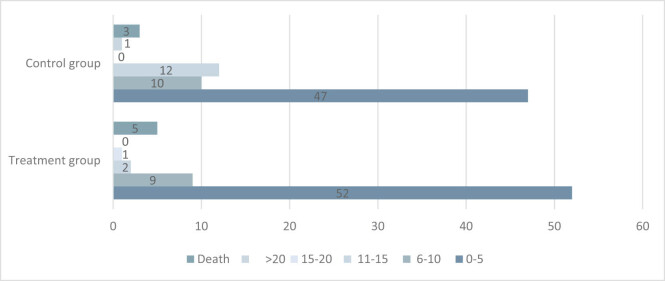
Contrast of NIHSS posttherapy on the two groups.

The proportion of NIHSS points between the treatment group and the control group after 90 days is shown in Figure 1.

### 3.4 Comparison of changes in mRS between two groups

As shown in Table 4, there was a statistically significant difference in the proportion of mRS 0–1 points between the treatment group and the control group (*P* < 0.05). As the treatment progressed, the proportion of mRS 0–1 points between the treatment group and the control group gradually increased, but the proportion of 0–1 points in the treatment group was consistently higher than that in the control group. After treatment, the proportion of mRS 0–1 scores in the thrombolysis treatment group was significantly higher than that in the thrombolysis control group, and the difference was statistically significant (*P* < 0.05). After treatment, the proportion of mRS 0-1 points in the thrombolysis treatment group was significantly higher than that in the thrombectomy control group, and the difference was statistically significant (*P* < 0.05), indicating that EDV dexborneol can effectively improve the neurological prognosis of patients with acute anterior circulation LVO.

**Table 4:** Proportion of mRS 0–1 points

Group	*N*	Proportion of the mRS score of 0–1, *n* (%)		
		14 days	30 days	90 days
Treatment group (*n* = 69)	69	24 (34.78%)	36 (52.17%)	44 (63.77%)
Control group (*n* = 73)	73	15 (20.55%)	20 (27.40%)	37 (50.68%)
*P* value	/	0.008	0.004	0.012
Thrombolysis group (*n* = 24)	24	7 (29.17%)	12 (50.00%)	15 (62.50%)
Thrombolysis control group (*n* = 21)	21	7 (33.33%)	10 (47.62%)	10 (47.62%)
*P* value	/	0.687	0.695	0.015
Thrombectomy treatment group (*n* = 39)	39	12 (30.77%)	19 (48.72%)	24 (61.54%)
Thrombectomy control group (*n* = 40)	40	12 (30.00%)	19 (47.50%)	18 (45.00%)
*P* value	/	0.923	0.854	0.026

**Figure 2:**
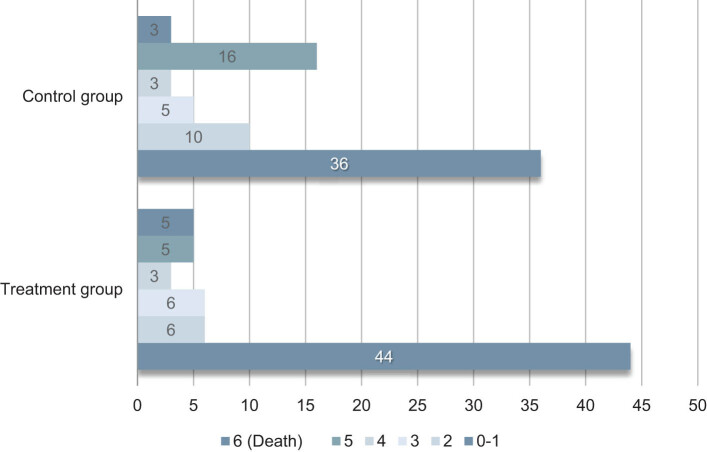
Contrast of mRS posttherapy on the two groups.

The proportion of mRS points between the treatment group and the control group after 90 days is shown in Figure 2.

### 3.5 Comparison of changes in inflammatory factors between two groups

Before treatment, there was no statistically significant difference in inflammatory factors between the two groups (*P* > 0.05). After treatment, the average levels of IL-6 and CRP in the treatment group were lower than those in the control group, and the difference was statistically significant (*P* < 0.05). The results are shown in Table 5.

**Table 5:** Statistical table of inflammatory factors

Group	On admission	Days 5–7	On days 10–14
Treatment group (IL-6)	33.6 ± 57.9	21.16 ± 29.25	5.864 ± 4.758
Control group (IL-6)	35.4 ± 99.5	21.51 ± 26.38	10.58 ± 16.54
*P* value	0.897	0.940	0.022
Treatment group (CRP)	27.23 ± 41.44	35.4 ± 99.5	11.43 ± 14.38
Control group (CRP)	25.24 ± 37.27	39.42 ± 46.41	19.67 ± 32.94
*P* value	0.868	0.839	0.044

### 3.6 Comparison of HT

In the study, the incidence of hemorrhagic transformation was significantly reduced in patients who underwent EDV dexborneol. Subgroup analyses for the thrombolysis group and endovascular thrombectomy group, the incidence of HT was also significantly reduced in study group with a statistically significant difference (*P* < 0.05), as shown in Table 6.

**Table 6:** Statistical table of hemorrhagic transformation

Group	*N*	Hemorrhagic transformation, *n* (%)	*P* value
Treatment group	69	14 (20.29%)	0.0006
Control group	73	29 (39.73%)	
The thrombolysis group	24	3 (12.50%)	0.0001
The thrombolysis control group	21	7 (33.33%)	
The thrombectomy group	39	10 (25.64%)	0.0001
The thrombectomy control group	40	19 (47.50%)	

## 4 Discussion

Acute ischemic stroke is a neurological impairment caused by sudden occlusion of both intracranial and extracranial arteries. In the first few hours after an ischemic attack, there is a salvageable ischemic penumbra around the core infarct area. Therefore, time is the brain, and opening blood vessels as soon as possible within the time window is the key to treat AIS. Therefore, vascular recanalization has become the standard treatment method for acute cerebral infarction. Thrombolysis has proven to be a significant advancement [15], but the recanalization rate of thrombolysis in different central veins varies from 10 to 33% due to the influence of thrombus location, thrombus load, etc. [16], and there is a risk of bleeding conversion, which is significantly time dependent and limits the beneficiary. According to the 2011 study, less than 5% of stroke patients received rt-PA thrombolysis treatment [17]. Intravascular thrombectomy therapy can effectively improve the functional prognosis of AIS patients caused by LVO and reduce mortality. However, some patients’ neurological function has not been effectively improved after good vascular recanalization, which is mainly related to the aggravation of neurological damage caused by ischemia–reperfusion injury [18]. Currently, the pathological and physiological processes of ischemia and reperfusion injury are complex and the mechanisms are not clear. The main pathological processes including energy metabolism disorders around the infarction, excitatory amino acid toxicity, neuroinflammation, oxidative stress, etc., ultimately mediate neuronal death [19]. Research has confirmed that rebuilding blood flow in ischemic tissue can lead to increased release of pro-inflammatory cytokines [20], leading to a series of pathological cascade reactions, directly or indirectly leading to cell apoptosis, disruption of the blood–brain barrier (BBB), brain edema, and HT. Meanwhile, oxidative stress is closely related to the mechanism of neuroinflammation, ultimately significantly promoting ischemia–reperfusion injury [21,22,23]. Early administration of antioxidants after stroke not only increases cell survival but also inhibits the inflammatory response induced by acute ischemia [24]. Interleukin-6, one of the main cytokines produced by the central nervous system [25], has been proven to have pro-inflammatory, prooxidative, and thrombogenic effects [26,27]. Clinical studies have shown that plasma IL-6 levels increase in patients with TIA or AIS, and plasma IL-6 concentration can predict the severity and clinical prognosis of stroke [28]. Therefore, at different stages of infarction, multiple pathological mechanisms are involved, and single treatment measures or drugs targeting a single mechanism are difficult to effectively control the disease. Treatment measures that eliminate free radicals and inhibit inflammatory reactions are suitable supplements for reperfusion injury after thrombolysis or intravascular treatment [8,9].

EDV dexborneol is a Class I innovative drug launched in China in 2020. It is composed of EDV and dexborneol in a 4:1 ratio and can effectively improve the functional prognosis of AIS patients [29]. To evaluate the efficacy of EDV dexborneol in patients with acute anterior circulation LVO, we enrolled 142 patients. The data of this study showed that after treatment, the NIHSS score and mRS score of the treatment group were significantly improved, better than the control group, and had a higher overall effective rate. Compared with the control group, the inflammatory factors IL-6 and CRP in the study group were lower than those in the control group after treatment. In this randomized clinical trial, EDV dexborneol can significantly improve the distribution of 90-day disability among patients with AIS due to anterior circulation LVO. To explore its mechanism, previous studies have shown that EDV is an antioxidant and free radical scavenger that can eliminate free radicals and inhibit endothelial cell damage, brain edema, and delayed neuronal death, thereby improving the functional outcomes of acute cerebral infarction. Borneol has anti-inflammatory effects and can inhibit the expression of inflammatory cytokines. At the same time, borneol can promote the penetration of EDV through the BBB and enhance the distribution of the drug in brain tissue [30]. The two components synergistically enhance the effect, blocking the mutual circulation between free radicals and inflammation [7,31,32]. In addition, ischemia–reperfusion injury can induce IL-6, increase iron overload, and induce iron death, And borneol can inhibit the expression of IL-6 [33], thereby exerting effective neuroprotective effects.

HT is a complication that may cause neurological deterioration in patients with AIS, particularly in LVO stroke. The overall rate of radiographic intracranial hemorrhage was 30% (42/142) in this trial, which was consistent with those of previous studies, which reported rates of 22–49% [34,35]. In patients receiving EDV dexborneol, a significantly decreased risk of radiographic intracranial hemorrhage was found compared with the control group. In subgroup analyses, patients in the thrombolysis group and thrombectomy group also had a numerically lower incidence of intracranial hemorrhage. The study’s finding of the potential benefit of EDV dexborneol in patients with ischemic stroke due to LVO may merit a future confirmatory trial confined to this population.

## 5 Conclusion

In summary, EDV dexborneol can improve the neurological prognosis of patients with acute anterior circulation LVO, reduce the levels of inflammatory factors IL-6 and CRP, and reduce bleeding conversion. It can be an effective supplement to thrombolysis and thrombectomy treatment. However, the data reported in this study require further validation due to the small sample size and investigation of a single-center study. Also, the follow-up was relatively short. In the future, we will continue to conduct relevant research to clarify the medium to long-term efficacy of EDV dexborneol therapy in patients with acute anterior circulation LVO.
